# Emerging Roles for Transcription Factors During Mitosis

**DOI:** 10.3390/cells14040263

**Published:** 2025-02-12

**Authors:** Samuel Flashner, Jane Azizkhan-Clifford

**Affiliations:** Department of Biochemistry and Molecular Biology, Drexel University College of Medicine, Philadelphia, PA 19102, USA

**Keywords:** transcription factor, mitosis, chromosome segregation, chromosome condensation, aneuploidy, genome instability, centromere, centrosome, kinetochore, chromosomal instability

## Abstract

The genome is dynamically reorganized, partitioned, and divided during mitosis. Despite their role in organizing interphase chromatin, transcription factors were largely believed to be mitotic spectators evicted from chromatin during mitosis, only able to reestablish their position on DNA upon entry into G_1_. However, a panoply of evidence now contradicts this early belief. Numerous transcription factors are now known to remain active during mitosis to achieve diverse purposes, including chromosome condensation, regulation of the centromere/kinetochore function, and control of centrosome homeostasis. Inactivation of transcription factors during mitosis results in chromosome segregation errors, key features of cancer. Moreover, active transcription and the production of centromere-derived transcripts during mitosis are also known to play key roles in maintaining chromosomal stability. Finally, many transcription factors are associated with chromosomal instability through poorly defined mechanisms. Herein, we will review the emerging roles of transcription factors and transcription during mitosis with a focus on their role in promoting the faithful segregation of sister chromatids.

## 1. Introduction

The adult human body contains approximately 30 to 37 trillion cells, each of which is derived from a single cell. The formidable expansion from a single progenitor requires an estimated 10 quadrillion cell divisions, during which each cell’s genomic information is duplicated and then faithfully divided into two daughter cells during mitosis [1,2]. Errorless chromosome segregation is indispensable for development and tissue homeostasis. Chromosomes are segregated by the complex collaboration between the microtubule spindle and mitotic chromosomes. Unsurprisingly, dysregulation and mutation of mitotic spindle components or factors that mediate mitotic assembly are associated with a variety of diseases, including cancer. Characterizing the molecular mechanisms regulating mitotic spindle assembly and function is therefore critical for unraveling the drivers of these diseases caused by chromosome segregation errors.

Historically, transcription factors were thought to be bystanders during mitosis. However, recent evidence has implicated these factors as crucial mediators of chromosome segregation through diverse targets, including mitotic chromosome condensation and kinetochore assembly (Table 1). In this review, we will summarize these emerging roles for transcription factors in chromosome segregation and discuss the implications of these findings.

### 1.1. Brief Overview of Chromosome Segregation

Chromosomes are segregated during mitosis through a tightly spatially and temporally regulated process. Mitotic progression is divided into six stages: prophase, metaphase, anaphase, telophase, and cytokinesis. Each stage contributes to the proper assembly, alignment, and segregation of sister chromatids and is therefore essential for the faithful transmission of genetic material. During prophase, chromosomes condense via the coordinated activity of the condensin complex proteins, and the nascent microtubule spindle begins to emanate from centrosomes. In prometaphase, proteinacious microtubule attachment points known as kinetochores mature at the centromere. The microtubule spindle attaches to these kinetochores and chromosomes begin to congress towards the spindle equator. During metaphase, condensed chromosomes are aligned along the metaphase plate. Microtubule/kinetochore attachments are surveilled by the spindle assembly checkpoint (SAC) to ensure that each chromosome pair is accurately connected to opposite poles of the mitotic spindle. Following satisfaction of the SAC, cells progress into anaphase in which each sister chromatid pair is separated by the removal of the cohesin complex proteins by the anaphase-promoting complex/cyclosome (APC/C). During telophase, the separated sister chromatids are divided equally across the two spindle poles, where they decondense and recruit the nuclear envelope. Finally, the cell undergoes cytokinesis, during which the cytoskeleton physically divides the cell into two by creating a cleavage furrow through contractile force. If completed correctly, two identical daughter cells enter G_1_ simultaneously having faithfully replicated their genomes.

In this review, we will focus on key elements of chromosome segregation regulated by mitotic transcription and transcription factors. Namely, mitotic chromosome condensation, centromere homeostasis, and centrosome homeostasis are all directly influenced by transcription factors. By collating these recent studies herein, we argue that transcription factors are key mediators of chromosome segregation, a process integral to human health and disease.

### 1.2. Chromosomal Instability (CIN) and Cancer

Dysfunction of the mitotic machinery results in chromosome missegregation, a key feature of cancer that is detectable in up to 90% of solid tumors. These errors contribute to the malignancy of the disease and are associated with poor patient prognosis, increased metastasis, and multidrug resistance [26,45]. Despite the pervasiveness and severity of these errors, their precise causes and consequences are still incompletely understood. Further, these errors appear to be cancer specific, as the missegregation rate of specific chromosomes in nontransformed cell lines is approximately 0.025% [46]. Therefore, research into the factors that regulate chromosome segregation has an immense potential impact on exploiting a cancer-specific vulnerability.

Chromosomal instability (CIN) is an elevated rate of chromosome missegregation throughout successive cell divisions. These errors are derived from defective chromosome segregation machinery that results in a loss of mitotic fidelity. Populations of CIN cells are therefore karyotypically diverse, which promotes intratumoral heterogeneity and its associated ramifications [47]. Subsequently, the factors that induce CIN are highly clinically relevant. Recent evidence has implicated the mitotic activity of transcription factors as a key process preventing CIN. Following a PubMed search of the terms “Chromosomal instability” and “transcription factor”, we identified a large number of factors associated with CIN in a variety of diseases (Table 1). Of these factors, many are known to localize to elements of the bipolar spindle during mitosis. Intriguingly, many of these factors localize to mitotic chromatin. However, the precise role of many of these factors during mitosis remains unclear. This knowledge gap is largely due to experimental limitations, which will be discussed in the following sections.

### 1.3. Historic Overview of Transcription Factors During Mitosis

Transcription factors were long believed to be evicted from mitotic chromatin. During mitosis, the chromosomes condense, and transcriptional programs were thought to cease [48,49]. Additionally, the conformation of mitotic chromosomes changes and the long-range enhancer–promoter contacts are eliminated [50]. Further, individual transcription factors were also mostly demonstrated to be evicted from the chromatin. One of the earliest studies that described this phenomenon determined that the ubiquitously expressed mammalian transcription factor Sp1 is removed from the hsp70 gene promoter [51]. Later studies confirmed that both specific transcription factors and components of the basal transcriptional machinery were removed from mitotic chromosomes [52]. Therefore, transcription factors were believed to have little direct role in mitotic progression due to the changes in the chromatin state, the cessation of transcription, and the removal of transcription factors from their promoters.

However, more recent evidence suggests that transcription factors can be retained at mitotic chromosomes (Table 1). Several recent studies have demonstrated that there is widespread retention of a diverse pool of transcription factors, including Sp1 [6,53,54,55,56,57,58]. These studies mostly evaluated transcription factor localization to mitotic chromatin using live-cell imaging in place of immunofluorescent detection in formaldehyde-fixed cells, suggesting that the earlier findings regarding transcription factor eviction were the result of a fixation artifact [55]. However, in certain cases, formaldehyde fixation does not prevent the detection of transcription factors at mitotic chromatin [59,60]. Therefore, this fixation artifact is not a universal barrier to detecting these mitotic associations. Regardless, there is now widespread acceptance that transcription factor binding is observed throughout mitosis. This retention is widely attributed to mitotic bookmarking, or the maintenance of transcriptional programs into G_1_, despite the dramatic reorganization of chromosomes during mitosis. In this model, transcription factors remain bound to the chromatin through mitosis to rapidly reactivate their transcriptional programs upon entry into G_1_. For example, the zinc finger transcription factor GATA1 is retained at key hematopoietic gene promoters during mitosis in mature erythroid cells [53]. Mitotic degradation of GATA1 in these cells reduced the re-expression of the hematopoietic genes upon entry into G_1_ and promoted the expression of immature cell markers [53]. Therefore, in certain cases, specific transcription factors can remain bound through mitosis to shepherd their transcriptional programs into G_1_. This phenomenon has been extensively reviewed elsewhere and will not be the focus of this work [61,62,63,64].

Recent evidence also suggests that most transcriptional programs remain intact during mitosis. For instance, more general transcription factors are retained at mitotic chromatin. TBP remains globally bound to promoters in mouse embryonic cells during mitosis [65]. Further, the same study determined through single molecule tracking in live cells that 15% of RNA pol II molecules remain bound to mitotic chromosomes compared to 30% of RNA pol II molecules during interphase. In line with these data, new evidence suggests that transcription is not globally suppressed during mitosis and that the cell’s transcriptional program is largely retained, albeit at lower levels, during mitosis [66]. Therefore, both transcription factors and their transcriptional programs are largely retained at a lower level during mitosis. Importantly, this transcription is integral to mitotic fidelity: inhibiting mitotic transcription is sufficient to induce chromosome segregation defects [67,68]. These findings provide key evidence highlighting the role of transcription factors in mitotic fidelity.

In summary, early experimental limitations led to the belief that transcription factors are bystanders during mitosis. However, recent studies have demonstrated that transcription factors are retained at mitotic chromatin; transcriptional programs remain active; and mitotic transcription is required for the faithful segregation of sister chromatids. This review will consider the mechanisms and implications of these recent studies to propose a novel paradigm that transcription factors are key mediators of mitotic fidelity, an essential process in human health.

### 1.4. Separating Mitotic Function of Transcription Factors from Their Transcriptional Programs

Separating the acute role of transcription factors during mitosis from their transcriptional programs is a major challenge that has limited progress in this field. Since transcription factors regulate the expression of diverse gene programs, loss of transcription factor function may dysregulate the expression of key mediators of chromosome segregation. Therefore, loss-of-function techniques that slowly ablate transcription factor protein levels over several cell cycles, such as RNAi or CRISPR knockout, are poor tools to separate the acute mitotic role of transcription factors from the indirect effects of their transcriptional programs. The ideal tool would deplete transcription factor protein levels immediately prior to mitosis and thereby would separate the mitotic function of the transcription factor from its downstream transcriptional targets.

Several emerging technologies have enabled progress in this field. Inducible degrons are small protein tags that promote the rapid (<1 h) degradation of proteins by targeting them to the proteasome in the presence of a small molecule, such as the plant hormone auxin [69]. Other technologies, such as proteolysis targeting chimeras (PROTACS), similarly induce the rapid proteosomal degradation of a target protein in response to a small molecule chimera [70,71]. Antibody-based methods, such as Trim-Away, can target a protein to the proteasome [72].

Implementing these technologies is resource intensive. Degron-based technologies require tagging the protein of interest prior to experimentation. Functional and soluble PROTACS are difficult to develop. Trim-Away requires microinjection or electroporation, which may alter cellular activity prior to mitosis. These limitations act as a moat, preventing their application to identify the acute role of transcription factors during mitosis.

In this review, we will focus on the direct and indirect evidence that transcription factors have acute roles during mitosis in regulating chromosomal segregation. Our goal is to provide the evidence justifying the resources required to specifically measure the mitotic activity of these factors. These experiments are integral to unraveling the mechanisms promoting CIN, a key feature of cancers.

## 2. Transcription Factors and Chromosome Condensation

Chromosome condensation promotes mitotic fidelity by preventing chromosome entanglement and supporting accurate formation and surveillance of the bipolar spindle. Almost 50 years ago, pioneering studies determined that chromosomes are condensed via DNA looping by scaffolding proteins into higher-order mitotic chromosomes [73,74,75]. The molecular machines that perform this reorganization, termed condensin complexes, were characterized in the ensuing decades [76,77,78,79,80]. More recent studies focused on the mechanism of action underlying condensin-mediated genome organization, as well as the regulation of these complexes. Unsurprisingly, loss of chromosome condensation is associated with chromosome segregation errors. Recent evidence has implicated transcription itself and transcription factors to be key mediators of this process vital to chromosomal stability.

### 2.1. Overview of Chromosome Condensation

In higher eukaryotes, chromosome condensation is mediated by condensin complexes I and II, which compact mitotic chromosomes through loop extrusion, during which DNA is moved through the ring complexes in an ATP-dependent manner [81,82,83,84,85,86]. The two complexes reorganize mitotic chromosomes through distinct yet complementary mechanisms. Recent models suggest that condensin complex II produces large loops of 200–400 DNA base pairs (bps), which are then organized into smaller (~80 bp) loops by condensin complex I [87,88]. This action results in axial compaction of the chromosome by condensin complex II and lateral compaction of the chromosome by condensin complex I. Thus, the two condensin complexes collaborate to fully compact mitotic chromosomes required for accurate segregation of sister chromatids.

Condensin complexes I and II are distinctly regulated through a variety of factors (reviewed in [89]), including spatiotemporal localization and posttranslational modification by mitotic kinases (e.g., CDK1 or AURKB). Recent evidence, which will be explored later in this section, has implicated transcription and transcription factors as key mediators of condensin complex function.

### 2.2. Chromosome Condensation Is Required for Chromosomal Stability

Condensin defects are, therefore, associated with chromosome segregation errors and result in the formation of ultrafine bridges and micronuclei [90,91]. Dysregulation and mutation of condensin complex protein gene expression are associated with aneuploidy in a variety of cancers, including lymphoma, colorectal, breast, lung, and ovarian [92,93,94,95,96]. Condensin defects are also associated with aneuploidy in developmental disorders, such as microcephaly [91]. Understanding how chromosome condensation regulates chromosome segregation is, therefore, highly clinically relevant.

Chromosome condensation promotes mitotic fidelity through a variety of mechanisms. DNA replication generates sister chromatid catenates, which can result in ultrafine DNA bridges and chromosome segregation errors if left unresolved during mitosis [90,97]. Condensin, in collaboration with topoisomerase II, decatenates these interlocked structures to allow for the resolution of sister chromatids in yeast [98], flies [99,100], frogs [101], and humans [102]. Chromosome condensation is also required for centromere function and surveillance of spindle assembly. During mitosis, microtubules stochastically attach to chromosomes at the kinetochore. Correctly attached microtubules are stabilized through a tension-dependent mechanism [103,104]. Condensin complex proteins help generate this tension and are, therefore, required for proper chromosome segregation [105,106]. Condensin-deficient yeast lose tension in the centromere, arrest in metaphase, and activate their spindle assembly checkpoint (SAC) [107]. In humans, condensin I is required for the centromere stiffness that is required for the biorientation of sister chromatids [105,108,109,110,111]. Therefore, condensin complexes are required to generate the tension required for correct microtubule attachment and thus mitotic fidelity. Parallel to this role, condensin complex II is required for the deposition of CENP-A, the key epigenetic determinant of the centromere [112,113]. In yeast, condensin deficiency results in the loss of Cse4p (CENP-A homolog) levels at the centromere. Together, chromosome condensation is an evolutionarily conserved and essential process ensuring that sister chromatids are faithfully divided during mitosis.

### 2.3. Transcription and Condensin Loading

Condensin complexes are enriched at highly expressed regions of DNA in a variety of model organisms, suggesting that transcription may promote condensin loading. In yeast, condensins bind to regions of ssDNA that are actively transcribed by RNA Polymerase II (RNAPII) [114]. Condensins are also enriched at mitotically upregulated and actively transcribed genes in yeast [115]. In chickens, condensin complex I localizes to the promoter regions of actively transcribed genes during mitosis to perform an unknown function [116]. In mice, condensin complex II is recruited to gene regulatory elements during interphase [117]. Together, these data indicate that condensin is recruited to areas of active transcription in yeast and vertebrates and that this interaction is required for proper chromosome segregation.

However, there is evidence that transcription per se may be insufficient or even deleterious to condensin-mediated compaction of mitotic chromosomes. Rapid depletion of RNAPII does not alter steady-state levels of condensin on mitotic chromosomes in fission yeast [118]. Further, transcription slows condensin translocation and loop extrusion in bacteria and yeast [118,119]. These findings raise the possibility that interactions between condensin complex proteins and transcription factors themselves may explain why condensins are enriched at highly or actively transcribed genes.

### 2.4. Transcription Promote Chromosome Condensation

Supporting the potential role of transcription factors as the driver of condensin localization to sites of active transcription, condensin complex genes frequently interact with transcription factors, and loss of transcription factor activity results in chromosome condensation defects and segregation errors. We were the first to demonstrate that a transcription factor is required for chromosome condensation in human cells [36]. The ubiquitously expressed transcription factor Sp1 promotes the loading of condensin complex I. Rapid depletion of Sp1 immediately prior to mitotic entry results in chromosome condensation defects, impaired mitotic progression, chromosome misalignment during metaphase, and increased micronuclei. How Sp1 is specifically regulating chromosome condensation is unclear but may be related to disrupted transcription through the centromere (Section 3) and/or impaired AURBK activity. Studies in other model organisms corroborate our findings that transcription factors mediate condensin complex loading and chromosome condensation. Yeast Cnd2 (CAP-H homolog) interacts with TATA box-binding protein (TBP) [120], which is required for condensin complex recruitment to mitotic chromosomes as well as proper chromosome segregation. Further, condensin is recruited to regions of DNA bound by transcription factors Ace2 and Ams2 during mitosis in yeast [3]. In vertebrates, Ncaph2 binds to regions occupied by transcription factor TFIIIC in mitotic mouse cells [121]. However, the authors did not determine if Ncaph2 was interacting with TFIIIC or if this similar localization pattern had any impact on cellular behavior. TFIIIC is known to interact with all condensin complex II proteins during interphase in mouse cells [40]. While this interaction supports the expression of several gene clusters, its role in mitotic chromatin organization remains unknown. Finally, in extracts prepared from human cells, CAP-G binds to TBP; however, the functional significance of this interaction is unknown [122]. Many transcription factors, including Sp1, have been shown to interact with other general transcription factors [123], which raises the possibility that interactions between general and specific transcription factors themselves may explain why condensins are enriched at highly or actively transcribed genes. Future studies should address this possibility.

Testing the hypothesis that transcription factors, rather than transcription, are required for condensin function is challenging due to difficulties separating the two. A recent study in *Xenopus* egg extracts, which are not transcriptionally active, overcame this limitation. In these extracts, the general transcription factor complex TFIIH is continuously required for chromosome condensation and localization of condensin complexes I and II [39]. These results suggest that transcription factor presence is more important to condensin loading than active transcription.

Ultimately, there is growing evidence implicating transcription factors in condensin-mediated chromosome organization. Mechanistically, how transcription factors influence this phenomenon is unclear and merits further study. Current models include recruitment through direct interaction [3,120,122], influencing histone occupancy and chromosome compaction [39], and modulation of apical regulators, such as AURKB [36]. Regardless of the mechanism, the depletion of these transcription factors results in loss of chromosome condensation and increased chromosome segregation errors and is, therefore, highly clinically relevant.

## 3. Transcription Factors and Centromeric Transcription

The kinetochore is integral to mitotic fidelity, serving as the chromosomal attachment point and site of surveillance for microtubules. The kinetochore is anchored to mitotic chromosomes at the centromere, a highly repetitive region of DNA. Recent evidence has implicated centromeric transcription as a key mediator of kinetochore assembly and function. The act of transcription itself, as well as the generation of unique lncRNAs from the centromere, are required for kinetochore homeostasis and cell division. We will discuss centromere and kinetochore biology with a focus on mitotic transcription regulation of these areas in this section.

### 3.1. Overview of Centromere and Kinetochore Assembly and Function

The kinetochore is the proteinacious structure that serves as the attachment point for the mitotic spindle and is, therefore, essential for chromosome segregation. The kinetochore comprises two regions: the inner kinetochore and the outer kinetochore. The inner kinetochore is organized via the constitutive centromere-associated network (CCAN), which consists of 16 subunits that are localized to centrochromatin throughout the cell cycle [124,125,126,127]. The CCAN performs diverse functions during the cell cycle, including acting as the interface between the kinetochore and the centromere, assembling the outer kinetochore, resisting the force generated by the mitotic spindle, and maintaining centromere identity following cell division [128,129,130,131]. Through these mechanisms, the CCAN and the inner kinetochore are essential for mitotic fidelity.

The outer kinetochore is dynamically assembled during the cell cycle and serves as the attachment point for microtubules as well as provides a platform for the mitotic checkpoint. The outer kinetochore is primarily composed of the Knl1 complex, the Mis12 complex, and the Ndc80 complex, which together comprise the 10-subunit KNM network required for microtubule attachment to mitotic chromosomes [132,133]. This network is mitosis specific and is rapidly assembled (<20 min) after mitotic entry [134,135]. In addition to anchoring microtubules, the KMN is a key substrate in the SAC (Section 1.1). AURKB is confined to the inner centromere but can phosphorylate substrates in the outer kinetochore KMN network in the absence of the tension generated by the bipolar spindle [136]. This differential phosphorylation creates a gradient of microtubule-binding affinity; full phosphorylation of these components, including the microtubule-binding protein NDC80, ablates microtubule binding [137,138]. However, proper spindle attachment to the kinetochore results in maximal tension generated by the bipolar spindle, which pulls the KMN network away from Aurora B, reducing the phosphorylation of KMN network substrates and, therefore, stabilizing correct microtubule attachments. Together, the inner and outer kinetochore are essential mediators of mitotic fidelity by serving as the attachment point and key point of regulation for microtubules.

The kinetochore is assembled at the centromere, a unique region of heterochromatin essential for mitotic fidelity. Human chromosomes contain monocentric centromeres that are defined epigenetically by the presence of the histone H3 variant CENP-A [139,140,141,142]. CENP-A is embedded in highly repetitive regions of AT-rich DNA sequences termed α-satellite DNA in humans [143,144]. These α-satellite repeats are flanked by repetitive regions of heterochromatin known as the pericentromere [145]. Together, the core centromere and pericentromere can be kilobases to megabases long [144,146]. Intriguingly, the sequences of both the core centromere and the pericentromere vary across each individual chromosome and are poorly conserved across species [144]. This heterogeneity appears to have a role in promoting mitotic fidelity. Chromosomes with a low degree of centromeric DNA heterogeneity were missegregated at an elevated rate compared to chromosomes with a higher degree of heterogeneity [147]. While the exact mechanisms underlying these findings are unclear, we will review the recent evidence that has implicated transcription of these regions during mitosis as a key factor required for kinetochore function and mitotic fidelity.

### 3.2. Centromere Homeostasis Is Required for Chromosomal Stability

Defects in centromere or kinetochore structure result in chromosome segregation errors and aneuploidy due to uncorrected errors in microtubule–chromosome attachments. For example, CENP-A depletion results in a variety of chromosome segregation errors, including the formation of micronuclei and nuclear bridges and an increase in aneuploidy [125,148,149]. The knockout of the CCAN subunit CENP-C results in defective mitotic progression and chromosome segregation errors [150]. Finally, loss of KMN function results in lagging chromosomes and severe chromosome segregation defects [138,151,152]. Ultimately, loss of centromere and kinetochore integrity compromises the surveillance of microtubule attachment to mitotic chromosomes, resulting in chromosome segregation errors.

### 3.3. Centromeric Transcription Supports Chromosome Segregation

The centromere was long believed to be transcriptionally silent during all phases of the cell cycle. However, recent evidence has identified active RNAPII at the centromere in a variety of organisms [67,68,153,154,155]. This transcription may influence centromere homeostasis in two ways: (1) by directly influencing the local composition of the centromere by inducing remodeling or recruiting centromere/kinetochore factors, (2) by the interaction of specific transcription factors with general transcription factors to promote the recruitment of condensins at actively transcribed genes (as noted above), and (3) by producing long noncoding RNAs (lncRNAs) that can serve as scaffolds, facilitating the assembly of centromere/kinetochore co-factors (review [156]).

One such mechanism is the recruitment of the microtubule attachment surveillance machinery to the inner centromere [67]. RNAPII is recruited to unattached kinetochores by the SAC protein Bub1 and is required for the translocation of a key mediator of sister chromatid cohesion and bipolar spindle formation, Sgo1, to the inner centromere [157]. Transcriptional inhibition further reduces Sgo1 intensity at mitotic centromeres and increases centromeric cohesion defects [158]. Further, active RNAPII is enriched at the inner centromere during mitosis and is required for AURKB recruitment and activation, potentially through interaction with noncoding RNA transcripts produced from the region [68]. The inhibition of RNAPII in this system results in defective kinetochore-microtubule attachments, further demonstrating the importance of this transcription.

Mitotic transcription is also required for the preservation of centromere identity. Transcription during late mitosis into early G_1_ is required for stable incorporation of CENP-A with centromeric chromatin [159]. This deposition may be the direct result of an interaction with an α-satellite lncRNA [160]. Centromere transcription supports the deposition of other CCAN components. Inhibiting RNAPII transcription during mitosis resulted in decreased CENP-C chromosomal localization and increased lagging chromosomes [67]. Intriguingly, this activity may be chromosome specific. Transcripts originating from each individual chromosome are unique and bind directly to CENP-A, CENP-B, and CENP-C. Depletion of these transcripts results in decreased CENP-A or CENP-C deposition at the chromosomes of origin [161].

Ultimately, centromeric transcription during mitosis is required to maintain mitotic fidelity through diverse mechanisms. Both the act of transcription per se and the production of lncRNAs from the centromere alter centromere identity and function. These processes are vital to the accurate segregation of sister chromatids.

### 3.4. Regulators of Mitotic Transcription

Despite compelling evidence that mitotic transcription through the centromere is integral to preserving chromosomal stability, little is known about the upstream regulators of this process. Current thinking holds that the unique and permissive chromatin environment facilitates active transcription through the centromere, and dynamic alteration of this environment promotes the mitotic specificity of this action. What factors influence these changes are unclear, however.

The centromere contains a unique blend of histone modifications that are associated with active or repressive chromatin [162]. The histone modifications associated with transcriptionally active chromatin include H3K36me^2^, H3K4me^1/2^, H3K9ac, H4K5ac, and H4K12ac [162,163,164,165,166,167,168] (review [156]). Loss of these marks may be important for centromere transcription and identity. In a human artificial chromosome (HAC) model, disrupting H3K4me patterns by tethering LSD1 to the HAC centromere reduces centromeric transcription and CENP-A incorporation [166]. Similarly, disrupting H4 acetylation by tethering the histone deacetylase HAD-1 reduces RNAPII transcription and CENPA deposition on a HAC [164]. These studies demonstrate that the unique architecture of histone modifications greatly influences mitotic transcription and centromere biology.

How this architecture is maintained and what is recruiting the transcriptional machinery to the centromere during mitosis remain unclear. Complicating these efforts, no bonafide transcription factor has been implicated in this process. While we have demonstrated that the transcription factor Sp1 localizes to prophase centromeres and is evicted following metaphase, more work is needed to link Sp1 localization with transcriptional activity [36]. Future studies should carefully evaluate which factors are required for mitotic transcription.

Together, these data implicate centromere transcription as a key regulator of mitotic fidelity. Key unmet areas in this field are to define the factors that regulate centromeric transcription. Recent evidence has demonstrated that a variety of transcription factors localize to the centromere coincidentally with active centromeric transcription. Yet, no transcription factor has been directly implicated in the control of this fundamental process.

## 4. Transcription Factors and Centrosome Biology

Centrosomes are small organelles that serve as the main microtubule organizer of the cell. During mitosis, the centrosome nucleates the mitotic spindle required for proper segregation of sister chromatids. Loss of centrosome function results in chromosome segregation errors. Transcription factors have long been shown to localize to mitotic centrosomes, and the loss of these factors during mitosis leads to centrosomal dysfunction and CIN.

### 4.1. Overview of Centrosome Function

The centrosome contains two microtubule-based centrioles surrounded by a complex protein matrix known as the pericentriolar material (PCM). The PCM houses γ-tubulin ring complexes, which are the primary anchors and nucleators of the microtubules. Mitotic cells contain two centrosomes, which are positioned at opposite ends of the cell and anchor the bipolar spindle. Upon completion of mitosis, each daughter cell houses one centrosome, which duplicates during the subsequent cell cycle to regenerate the machinery required for bipolar spindle formation. Intriguingly, many transcription factors dynamically localize to the centrosome specifically during mitosis [169].

### 4.2. Centrosome Homeostasis Is Required for Chromosomal Stability

Defective centrosome function results in CIN through diverse mechanisms [170]. Supernumerary centrosome number (n > 2 during mitosis) can disrupt the bipolar spindle, promoting multipolar mitosis (segregation into n > 2 daughter cells) or pseudo-bipolar mitosis (in which sister chromatids are divided unequally into each daughter cell). Supernumerary centrosomes occur through centrosome over-duplication during the cell cycle or fragmentation during mitosis. Alternatively, centrosomes that are unable to nucleate the mitotic spindle can promote cytokinesis failure and tetraploidy [171].

Unsurprisingly, centrosome defects are associated with CIN in a variety of cancers, including breast [172], prostate [173], colon [174], gastric [175], lung [176], pancreas [177], and cervix [178] (review [179]). Inducing centrosome amplification is sufficient to induce aneuploidy and spontaneous tumorigenesis arising from a variety of tissues, including T- and B-cell lymphomas, squamous cell carcinomas, and sarcomas [180]. Therefore, understanding the key regulators of centrosome biology will provide clinically relevant mechanistic insights into a major driver of cancer.

### 4.3. Transcription Factors Support Centrosome Homeostasis

Many transcription factors localize to the centrosome during mitosis and regulate centrosome function through diverse mechanisms. Some transcription factors may regulate PCM composition and prevent the inappropriate fragmentation of centrosomes. Transcription factor p53 undergoes ATM-dependent localization to mitotic centrosomes [30,31]. Loss of centrosomal p53 results in centrosome fragmentation and amplification [32,33]. These changes can occur prior to malignant transformation. p53-associated centrosome amplification is detectable in pre-malignant Barrett’s esophagus and increases in dysplasia, malignant transformation, and metastasis [33]. These results highlight how transcription factor localization to mitotic centrosomes plays a key tumor suppressive role in preventing malignant transformation. Further, the transcription factor ATF5 regulates PCM composition, centriolar integrity, and bipolar spindle formation through localization to mitotic centrosomes [8,9]. ATF5 accumulates at mitotic centrosomes prior to its eviction via SUMO-ylation at the end of the M phase [8]. Blocking the SUMO-ylation of ATF5 results in inappropriate retention of ATF5 through the M phase, disrupted centrosome cycle, and genome instability [9]. Further, SNAP_45_, a component of the small RNA-activating protein complex SNAP_c_, regulates the transcription of small nuclear RNA genes and dynamically binds to centrosomes during mitosis [35]. Loss of SNAP_45_ results in supernumerary centrosomes and multipolar mitosis [35]. Rcd1, Rcd5, MBD-R2, and Wds, key regulators of housekeeping genes in *Drosophila*, localize to mitotic centrosomes [25]. Loss of these genes results in impaired centrosome duplication and chromosome segregation defects.

Other transcription factors alter microtubule dynamics. Phosphorylated YB-1 localizes to mitotic centrosomes [43]. Loss of YB-1 results in inappropriate microtubule detachment, defective nuclear envelope reassembly, cytokinesis failure, and aneuploidy [42,43,44]. In addition, OCT1 localizes to mitotic centrosomes following phosphorylation by NEK6 [28]. Loss of OCT1 results in abnormal tubulin staining, indicative of altered microtubule dynamics [28]. Finally, RXR-α localizes to the centrosome following CDK1-dependent phosphorylation [34]. Loss of RXR-α results in reduced PLK1 activity, decreased microtubule dynamics, altered centrosome maturation, and chromosome segregation defects [34].

In addition, transcription factors influence centrosome dynamics during interphase. We demonstrated that the transcription factor Sp1 localizes at centrosomes [37]. Loss of Sp1 results in increased centriole splitting, multipolar mitosis, and loss of microtubule nucleation [37]. Further, ARKNA is required for microtubule nucleation throughout the cell cycle [181]. Finally, SF1 is located in the centriole, and loss of SF1 results in centrosome over-duplication and chromosome segregation errors. These results demonstrate that transcription factor control of centrosomes is not confined exclusively to mitosis.

Together, these studies highlight how transcription factors regulate centrosome biology during mitosis, a key component of genome stability. Transcription factors regulate centrosome integrity, number, and control of microtubule dynamics, keys to maintaining chromosomal stability.

## 5. Discussion

**Figure 1:**
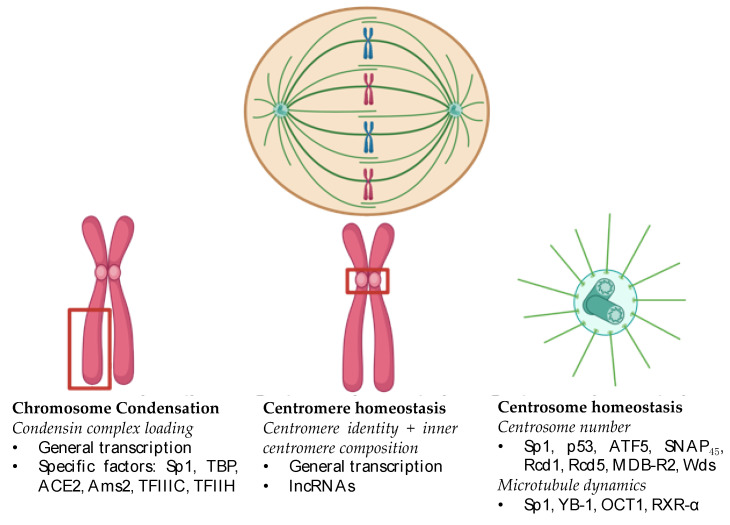
Overview of transcription factor control of chromosome segregation. Red boxes indicate condensed chromosomes (left) or centromere (center). Made with www.biorender.com.

Transcription factors directly regulate mitotic fidelity through diverse mechanisms, including maintenance of key elements of the mitotic spindle: chromosome condensation, centromere homeostasis, and the centrosome (Figure 1). Loss of transcription factor levels during mitosis disrupts chromosome segregation and results in CIN, a key feature of cancer (Table 1). Despite this highly clinically relevant function of transcription factors, elucidating their mitotic functions has been a slow process.

This knowledge gap is largely due to technological limitations in detecting transcription factor localization to mitotic chromatin and separating the acute mitotic functions of transcription factors from their transcriptional programs. However, recent progress in separating these functions has overcome some of these limitations, enabling progress in the field of mitotic transcription factor biology. We were the first to apply rapid degron technology to evaluate the acute roles of transcription factors during mitotic chromosome segregation. Through this approach, we demonstrated that the mitotic activity of Sp1 is required for chromosome segregation and condensation [36]. These findings provide proof of principle that utilizing such technologies can be fruitful. Others have performed similar approaches. Rapid degradation of AID-TOP1 results in a loss of RNAPII and chromosome segregation defects [182]. An additional study determined that rapid depletion of AID-tagged RNAPII (Rpb1-sAID) did not alter chromosome condensation in yeast [118]. This study further suggests that inappropriate retention of RNAPII may actually impede accurate condensin-mediated chromosome segregation during anaphase [118]. These findings highlight how careful application of rapid depletion of transcription factors is essential to unravel the complex mechanisms of transcription factor activity during mitosis. In addition, mitotic degradation of transcription factors YY1 [183], CTCF [184], SOX2 [185], ESRRB [186], NR5A2 [186], and TBD [65] has been performed to assess changes in mitotic bookmarking and chromatin organization. However, these studies did not evaluate chromosome segregation defects. Together, these studies demonstrate both the feasibility and need for rapid degradation studies to interrogate mitotic roles for transcription factors during chromosome segregation.

Unraveling the mechanisms mediating transcription factor control of mitotic segregation and CIN has a potential clinical impact. Therapeutic strategies that target CIN are an emerging area of precision medicine [187]. CIN can promote intratumoral heterogeneity, drug resistance, and metastasis; however, excess CIN results in cell death. Therefore, CIN+ cells are precariously balanced between death and a loss of their oncogenic properties. As a result, there are two primary strategies for targeting CIN: exacerbating CIN to induce cell death or reducing CIN to dampen its deleterious effects. Therapies that aim to potentiate CIN to lethal levels involve targeting centrosome clustering, microtubule de/stabilizers, and SAC inhibitors (reviewed thoroughly in [187]). These therapies can target the mitotic machinery (e.g., microtubules) as well as the underlying chromatin (e.g., HDAC inhibitors [188]). Conversely, reversing the deleterious effects of CIN involves correcting the mitotic defect caused by the loss of transcription factors. For example, stabilizing cohesins in pRB-deficient lung cancers reversed chromosome segregation errors, which is a potential chemopreventative strategy [189]. Together, these strategies highlight the clinical relevance of transcription factor control of chromosome segregation during mitosis.

Why transcription factors have such an outsized role during mitosis remains unclear. We have studied transcription factors moonlighting in other contexts, namely, the DNA damage response [190,191,192,193] and apoptosis [194]. We speculate that transcription factor localization to the chromatin and its ability to interact with a variety of proteins through its transactivation domains position transcription factors as ideal platforms for a variety of different cellular processes. During mitosis, transcription factor retention at the chromosomes brings it into proximity with the mitotic machinery (e.g., chromosome condensation factors, kinetochore proteins, and spindle regulators). Therefore, these proteins and factors are well positioned to evolve secondary functions [169]. Further, one intriguing possibility is that the loss of key transcription factors at mitotic chromosomes (Table 1) constitutes an existential threat to cell identity (e.g., through the loss of bookmarking) that is eliminated through mitotic defects, which are lethal in nontransformed cells. Another possibility is that the changes required to facilitate mitotic chromosome organization (e.g., permitting condensin access to the chromatin) are also highly similar to the changes required to facilitate transcription (e.g., permitting RNAPII access to the chromatin). Transcription factors are well suited to facilitate both of these chromatin changes. Future studies should explore these possibilities.

Together, transcription factors are crucial and underappreciated mediators of mitotic fidelity. Recent advancements in rapid protein degradation have catalyzed the recent progress in this field. However, there is more work to be performed, as few studies have applied these technologies to interrogate the mitosis-specific role of transcription factors in mediating chromosome segregation. Closing this knowledge gap will lead to a better understanding of the key mechanisms preventing CIN with strong therapeutic potential.

## Figures and Tables

**Table 1:** Transcription factors associated with CIN.

Factor	Evidence	Mitotic Localization	Citation
**ACE2**	Condensation defects	Chromosome enrichment	[3]
**Aft1**	Aneuploidy, Cohesin defects	Unknown	[4]
**Ams2**	Condensation defects	Chromosome enrichment	[3]
**AP4**	Micronuclei, Aneuploidy	Intermediate chromosome enrichment	[5,6]
**Atf3**	Aneuploidy, Micronuclei	Intermediate chromosome enrichment	[6,7]
**ATF5**	Centrosome amplification	Centrosome	[8,9]
**B-Myb**	Aneuploidy, Micronuclei, Condensation defects	Unknown	[10,11]
**CDX2**	Anaphase bridges	Chromosome enrichment	[6,12]
**CEBPD**	Aneuploidy	Unknown	[13,14]
**cMYC**	Micronuclei, Aneuploidy	Intermediate chromosome enrichment	[5,6,15,16]
**E2F**	Aneuploidy	Unknown	[17]
**EAP30**	Centrosome defects, Microtubule dysfunction	Unknown	[18]
**FOXM1**	Aneuploidy, Cytokinesis failure, CIN-related gene expression	Depleted at mitotic chromosomes	[6,19,20,21,22]
**GATA6**	Aneuploidy, Nuclear envelope defects, Cytokinesis failure	Unknown	[23]
**Ikaros**	Aneuploidy	Unknown	[24]
**MDB-R2**	Centrosome amplification	Centrosome	[25]
**NF-κB**	Centrosome amplification	Chromosome enrichment	[26,27]
**OCT1**	Altered microtubule dynamics	Centrosome	[28]
**ONECUT3**	Aneuploidy	Unknown	[29]
**p53**	Centrosome amplification	Centrosome	[30,31,32,33]
**RB1**	Aneuploidy	Unknown	[17]
**Rcd1**	Centrosome amplification	Centrosome	[25]
**Rcd5**	Centrosome amplification	Centrosome	[25]
**RXR-a**	Altered microtubule dynamics	Centrosome	[34]
**SNAP45c**	Centrosome amplification	Centrosome	[35]
**Sp1**	Aneuploidy, Micronuclei, Centrosome amplification, Condensation defects	Centromere	[36,37]
**SRF**	Aneuploidy, Condensation defects	Unknown	[38]
**TFIIH**	Condensation defects	Chromosome enrichment	[39]
**TFIIIC**	Condensation defects	Chromosome enrichment	[40]
**Twist1**	Aneuploidy	Unknown	[41]
**Wds**	Centrosome amplification	Centrosome	[25]
**YAP1**	CIN-related gene expression	Unknown	[22]
**YB-1**	Centrosome amplification, Cytokinesis failure	Unknown	[42,43,44]

## Data Availability

Not applicable.
